# Stratified wind from a super-Eddington X-ray binary is slower than expected

**DOI:** 10.1038/s41586-025-09495-w

**Published:** 2025-09-17

**Authors:** XRISM collaboration, Marc Audard, Hisamitsu Awaki, Ralf Ballhausen, Aya Bamba, Ehud Behar, Rozenn Boissay-Malaquin, Laura Brenneman, Gregory V. Brown, Lia Corrales, Elisa Costantini, Renata Cumbee, María Díaz Trigo, Chris Done, Tadayasu Dotani, Ken Ebisawa, Megan Eckart, Dominique Eckert, Teruaki Enoto, Satoshi Eguchi, Yuichiro Ezoe, Adam Foster, Ryuichi Fujimoto, Yutaka Fujita, Yasushi Fukazawa, Kotaro Fukushima, Akihiro Furuzawa, Luigi Gallo, Javier A. García, Liyi Gu, Matteo Guainazzi, Kouichi Hagino, Kenji Hamaguchi, Isamu Hatsukade, Katsuhiro Hayashi, Takayuki Hayashi, Natalie Hell, Edmund Hodges-Kluck, Ann Hornschemeier, Yuto Ichinohe, Manabu Ishida, Kumi Ishikawa, Yoshitaka Ishisaki, Jelle Kaastra, Timothy Kallman, Erin Kara, Satoru Katsuda, Yoshiaki Kanemaru, Richard Kelley, Caroline Kilbourne, Shunji Kitamoto, Shogo Kobayashi, Takayoshi Kohmura, Aya Kubota, Maurice Leutenegger, Michael Loewenstein, Yoshitomo Maeda, Maxim Markevitch, Hironori Matsumoto, Kyoko Matsushita, Dan McCammon, Brian McNamara, François Mernier, Eric D. Miller, Jon M. Miller, Ikuyuki Mitsuishi, Misaki Mizumoto, Tsunefumi Mizuno, Koji Mori, Koji Mukai, Hiroshi Murakami, Richard Mushotzky, Hiroshi Nakajima, Kazuhiro Nakazawa, Jan-Uwe Ness, Kumiko Nobukawa, Masayoshi Nobukawa, Hirofumi Noda, Hirokazu Odaka, Shoji Ogawa, Anna Ogorzalek, Takashi Okajima, Naomi Ota, Stephane Paltani, Robert Petre, Paul Plucinsky, Frederick Scott Porter, Katja Pottschmidt, Kosuke Sato, Toshiki Sato, Makoto Sawada, Hiromi Seta, Megumi Shidatsu, Aurora Simionescu, Randall Smith, Hiromasa Suzuki, Andrew Szymkowiak, Hiromitsu Takahashi, Mai Takeo, Toru Tamagawa, Keisuke Tamura, Takaaki Tanaka, Atsushi Tanimoto, Makoto Tashiro, Yukikatsu Terada, Yuichi Terashima, Yohko Tsuboi, Masahiro Tsujimoto, Hiroshi Tsunemi, Takeshi G. Tsuru, Aysegül Tümer, Hiroyuki Uchida, Nagomi Uchida, Yuusuke Uchida, Hideki Uchiyama, Yoshihiro Ueda, Shinichiro Uno, Jacco Vink, Shin Watanabe, Brian J. Williams, Satoshi Yamada, Shinya Yamada, Hiroya Yamaguchi, Kazutaka Yamaoka, Noriko Yamasaki, Makoto Yamauchi, Shigeo Yamauchi, Tahir Yaqoob, Tomokage Yoneyama, Tessei Yoshida, Mihoko Yukita, Irina Zhuravleva, Joey Neilsen, Ryota Tomaru, Missagh Mehdipour

**Affiliations:** 1 Department of Astronomy, University of Geneva, Versoix, Switzerland; 2 Department of Physics, Ehime University, Ehime, Japan; 3 Department of Astronomy, University of Maryland, College Park, MD USA; 4 NASA / Goddard Space Flight Center, Greenbelt, MD USA; 5 Center for Research and Exploration in Space Science and Technology, NASA / GSFC (CRESST II), Greenbelt, MD USA; 6 Department of Physics, University of Tokyo, Tokyo, Japan; 7 Department of Physics, Technion, Technion City, Haifa, Israel; 8 Kavli Institute for Astrophysics and Space Research, Massachusetts Institute of Technology, Cambridge, MA USA; 9 Center for Space Science and Technology, University of Maryland, Baltimore County (UMBC), Baltimore, MD USA; 10 Center for Astrophysics | Harvard-Smithsonian, Cambridge, MA USA; 11 Lawrence Livermore National Laboratory, Livermore, CA USA; 12 Department of Astronomy, University of Michigan, Ann Arbor, MI USA; 13 SRON Netherlands Institute for Space Research, Leiden, The Netherlands; 14 ESO, Karl-Schwarzschild-Strasse 2, 85748, Garching bei München, Germany; 15 Centre for Extragalactic Astronomy, Department of Physics, University of Durham, South Road, Durham, UK; 16 Kavli IPMU (WPI), UTIAS, The University of Tokyo, Kashiwa, Chiba, Japan; 17 Institute of Space and Astronautical Science (ISAS), Japan Aerospace Exploration Agency (JAXA), Kanagawa, Japan; 18 Department of Physics, Kyoto University, Kyoto, Japan; 19 Department of Economics, Kumamoto Gakuen University, Kumamoto, Japan; 20 Department of Physics, Tokyo Metropolitan University, Tokyo, Japan; 21 Department of Physics, Hiroshima University, Hiroshima, Japan; 22 Department of Physics, Fujita Health University, Aichi, Japan; 23 Department of Astronomy and Physics, Saint Mary’s University, Nova Scotia, Canada; 24 Cahill Center for Astronomy and Astrophysics, California Institute of Technology, Pasadena, CA USA; 25 European Space Agency (ESA), European Space Research and Technology Centre (ESTEC), Noordwijk, The Netherlands; 26 Faculty of Engineering, University of Miyazaki, Miyazaki, Japan; 27 RIKEN Nishina Center, Saitama, Japan; 28 Leiden Observatory, University of Leiden, P.O. Box 9513, NL-2300 RA, Leiden, The Netherlands; 29 Department of Physics, Saitama University, Saitama, Japan; 30 Department of Physics, Rikkyo University, Tokyo, Japan; 31 Faculty of Physics, Tokyo University of Science, Tokyo, Japan; 32 Faculty of Science and Technology, Tokyo University of Science, Chiba, Japan; 33 Department of Electronic Information Systems, Shibaura Institute of Technology, Saitama, Japan; 34 Department of Earth and Space Science, Osaka University, Osaka, Japan; 35 Department of Physics, University of Wisconsin, Madison, WI USA; 36 Department of Physics and Astronomy, University of Waterloo, Ontario, Canada; 37 Department of Physics, Nagoya University, Aichi Japan; 38 Science Research Education Unit, University of Teacher Education Fukuoka, Fukuoka, Japan; 39 Hiroshima Astrophysical Science Center, Hiroshima University, Hiroshima, Japan; 40 Department of Data Science, Tohoku Gakuin University, Miyagi, Japan; 41 College of Science and Engineering, Kanto Gakuin University, Kanagawa, Japan; 42 European Space Agency(ESA), European Space Astronomy Centre (ESAC), Madrid, Spain; 43 Department of Science, Faculty of Science and Engineering, KINDAI University, Osaka, Japan; 44 Department of Teacher Training and School Education, Nara University of Education, Nara, Japan; 45 Astronomical Institute, Tohoku University, Miyagi Japan; 46 Department of Physics, Nara Women’s University, Nara, Japan; 47 School of Science and Technology, Meiji University, Kanagawa Japan; 48 Yale Center for Astronomy and Astrophysics, Yale University, USA; 49 Department of Physics, Konan University, Hyogo, Japan; 50 Graduate School of Science and Engineering, Kagoshima University, Kagoshima, Japan; 51 Department of Physics, Chuo University, Tokyo, Japan; 52 Faculty of Education, Shizuoka University, Shizuoka, Japan; 53 Department of Astronomy, Kyoto University, Kyoto, Japan; 54 Nihon Fukushi University, Shizuoka, Japan; 55 Anton Pannekoek Institute, the University of Amsterdam, Amsterdam, The Netherlands; 56 RIKEN Cluster for Pioneering Research, Saitama, Japan; 57 Johns Hopkins University, Baltimore, MD USA; 58 Department of Astronomy and Astrophysics, University of Chicago, Chicago, IL USA; 59 Villanova University, Mendel Science Center 263B, Villanova, USA; 60 Space Telescope Science Institute, Baltimore, MD USA

**Keywords:** Compact astrophysical objects, High-energy astrophysics

## Abstract

Accretion disks in strong gravity ubiquitously produce winds, seen as blueshifted absorption lines in the X-ray band of both stellar mass X-ray binaries (black holes and neutron stars)^1–4^ and supermassive black holes^5^. Some of the most powerful winds (termed Eddington winds) are expected to arise from systems in which radiation pressure is sufficient to unbind material from the inner disk (*L* ≳ *L*_Edd_). These winds should be extremely fast and carry a large amount of kinetic power, which, when associated with supermassive black holes, would make them a prime contender for the feedback mechanism linking the growth of those black holes with their host galaxies^6^. Here we show the XRISM Resolve spectrum of the galactic neutron star X-ray binary, GX 13+1, which reveals one of the densest winds ever seen in absorption lines. This Compton-thick wind significantly attenuates the flux, making it appear faint, although it is intrinsically more luminous than usual (*L* ≳ *L*_Edd_). However, the wind is extremely slow, more consistent with the predictions of thermal-radiative winds launched by X-ray irradiation of the outer disk than with the expected Eddington wind driven by radiation pressure from the inner disk. This puts new constraints on the origin of winds from bright accretion flows in binaries, but also highlights the very different origin required for the ultrafast (*v* ~ 0.3*c*) winds seen in recent Resolve observations of a supermassive black hole at a similarly high Eddington ratio^7^.

## Abstract (web summary)

The XRISM Resolve spectrum of the galactic neutron star X-ray binary, GX 13+1, reveals one of the densest winds ever seen in absorption lines.

## Main

GX 13+1 is a disk-accreting neutron star in a 24.5-day orbit^8,9^ with a giant (K5 III) companion star^10^, which gives a large mass transfer rate through the Roche lobe^11^, resulting in a persistently bright X-ray source (*L* ≈ 0.5*L*_Edd_ for a 1.4*M*_⊙_ neutron star at a distance of 7 kpc; ref. ^10^). The presence of dips in the X-ray lightcurve indicates a high binary inclination^12^, which is optimal for observations of accretion disk winds^1,13,14^. Every X-ray observation of GX 13+1 with sufficient spectral resolution has shown blueshifted absorption lines^15–18^, making it an ideal target for Resolve, an X-ray micro-calorimeter onboard the new JAXA/NASA/ESA mission XRISM^19^. Resolve has an energy resolution of 4.5 eV at 6 keV, which is a factor of 4 better than the previous state-of-the-art equipment for bright binaries (third-order data^20^ from the High-Energy Transmission Grating Spectrometer^21^, hereafter HETGS), and with a much larger effective area, especially above 7 keV. The combination of improved resolution and larger area enables more sensitive measurements of the velocity and ionization structure of accretion disk winds, important for diagnosing the physical properties and launch mechanisms of these outflows^22^.

**Fig. 1:**
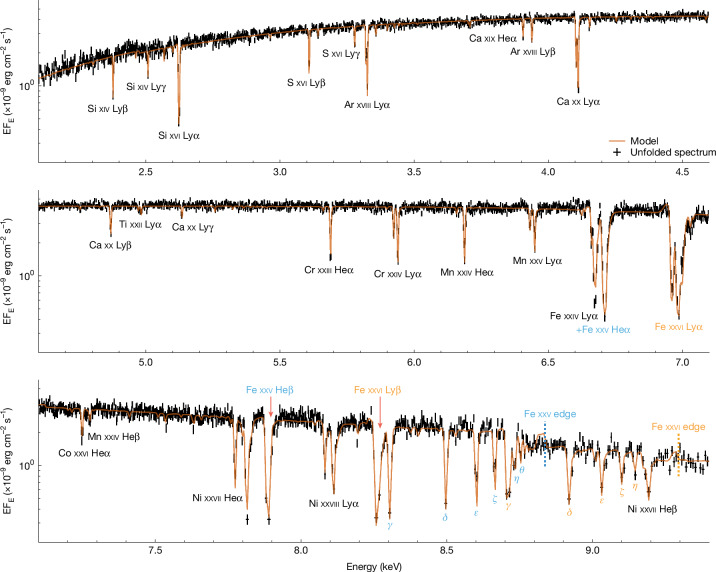
The Resolve/XRISM spectrum of GX 13+1. This is dominated by multiple absorption lines from H- and He-like ions, blueshifted by about 330 km s^−1^. All the fine structure transitions in these lines are resolved, showing that the lines are very narrow (velocity dispersion of around 150 km s^−1^). All strong lines are labelled; the 1 − *n* transitions of Fe xxv and Fe xxvi are indicated in cyan and orange, respectively. Even the weakest line identified here (Ti xxii Lyα_1,2_ around 4.95 keV) is highly significantly detected (Δ*χ*^2^ = 32 for 1 additional degree of freedom). The orange line shows the best-fit model described in the text, with an intrinsic continuum absorbed by the slow wind, but with a faster (700 km s^−1^), broader (300 km s^−1^) even more highly ionized component to fit the blue wing seen in Fe xxvi Lyα_1,2_ (Fig. 3). The model also includes diffuse emission from the wind (modelled using scattered intrinsic flux plus photoionized line and recombination continua from both wind components, all with some self-absorption in the wind). This fits the data fairly well overall (Methods and Extended Data Table 2), except around the Fe xxv (8.8 keV) and Fe xxvi (9.25 keV) edges, in which the photoionization model used here is incomplete (only including transitions up to *n* = 16). The total electron scattering optical depth in the slow wind is *τ*_es_ ~ 1, and both winds is *τ*_es_ ~ 1.8, attenuating the intrinsic flux (Fig. 2).

The new data on GX 13+1 from Resolve were taken on 25 February 2024; more details of the observations and data analysis are given in the Methods. The Resolve spectrum, shown in Fig. 1, shows dozens of strong, slightly blueshifted (*v*_out_ ≈ 330 km s^−1^), narrow (*v*_turb_ ≈ 150 km s^−1^) X-ray absorption lines. Most of these are from H-like and He-like ions, of multiple elements (Si, S, Ar, Ca, Ti, Cr, Mn, Fe, Co and Ni), indicating a highly ionized absorber.

Many of the lines below 7 keV (Fig. 1a,b) have been studied before, although at lower resolution and signal-to-noise ratios, in Chandra HETGS observations of this source^18^. What is completely new is the number and depth of lines above 7 keV (Fig. 1c), with multiple higher-order transitions detected out to at least K*θ* (*n* = 1–9) for Fe xxv and K*η* (*n* = 1–8) for Fe xxvi. These lines have small oscillator strengths, so their depth requires the column density of the wind to be extremely high, both in an absolute sense and in comparison with the previous Chandra observations (see below).

We first model the absorption lines from each ion separately and find that these are consistent with being produced by a single absorber with an ionization parameter log *ξ* ≈ 3.9 and an equivalent column density of *N*_H_ ≈ 1.3–1.4 × 10^24^ cm^−2^, assuming solar abundances. We confirm this by modelling all the ions together using the photoionization code PION (refs. ^23,24^) (Methods). The derived column is so large that the wind is optically thick for electron scattering, with *τ*_es_ ≈ 1. This attenuates the radiation from the central X-ray source as it passes through the wind, reducing the direct continuum flux by a geometry-dependent factor of exp(*τ*_es_) ≈ 3. Correcting for this effect, we infer a bolometric luminosity of *L* = 1.8 × 10^38^ erg s^−1^, which is approximately *L*_Edd_: this Compton-thick wind is produced by a source radiating at the Eddington limit.

**Fig. 2:**
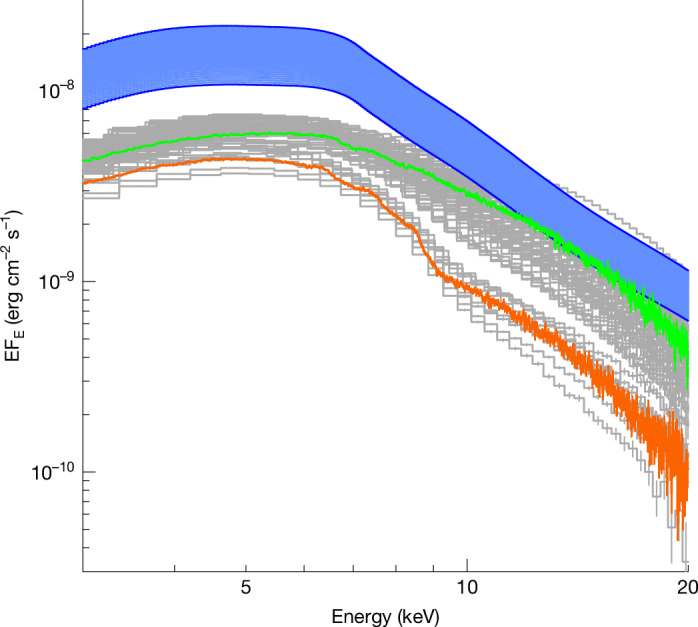
Historical X-ray variability of GX 13+1. The archival NuSTAR spectrum of GX 13+1 (green) is similar to most of the archival RXTE data (grey). Instead, the XRISM-coordinated NuSTAR spectrum (orange) has lower flux and shows a much deeper K-edge from Fe xxv at 8.8 keV. On closer inspection, 5–10% of the archival RXTE spectra are similar to this recent NuSTAR observation, indicating that this dense wind/super-Eddington phase is recurring in the source. The blue band shows a range of possible continuum spectra of GX 13+1 after correcting for attenuation due to electron scattering in the wind. The lower end of the envelope corresponds to *τ*_es_ = 1 from the slow wind alone, whereas the upper end corresponds to *τ*_es_ = 1.8 as inferred from the best-fit model for the slow plus fast wind. The source is intrinsically more luminous than normal, at or above Eddington.

Both these conclusions were initially surprising, as the ionized winds seen in previous observations of GX 13+1 with the Chandra HETGS had large but optically thin column densities^15,18^ of *N*_H_ ≈ 2–3 × 10^23^ cm^−2^, from a source with large but sub-Eddington luminosities of about 0.5*L*_Edd_. The unusual behaviour of GX 13+1 during our observation is illustrated by our simultaneous NuSTAR broadband X-ray data (Methods). These are shown as the orange points in Fig. 2, in which we also show an archival NuSTAR spectrum (green). GX 13+1 is noticeably fainter during our observation and has a much stronger absorption feature at around 8.8 keV, that is, at the K-edge of Fe xxv. The column density in this ion is much larger than in the majority of archival data. Other archival broadband datasets^25^ (RXTE, grey) confirm that this ‘reduced flux/strong Fe xxv edge’ state is very unusual (≲10% of observations), but lack the energy resolution to unambiguously connect this behaviour to attenuation in a high-column wind.

Correcting our best-fit continuum model for electron scattering attenuation in the slow wind gives an intrinsic flux shown as the lower edge of the blue band in Fig. 2, implying the source is intrinsically more luminous than normal. We suggest a causal relationship: an increase in the intrinsic X-ray luminosity enhances the wind to such an extent that it becomes Compton-thick, suppressing the observed flux and making the source appear dimmer (but with strong wind signatures). The increase in wind column may also explain the different X-ray polarization properties seen by IXPE during the XRISM observation^26^.

**Fig. 3:**
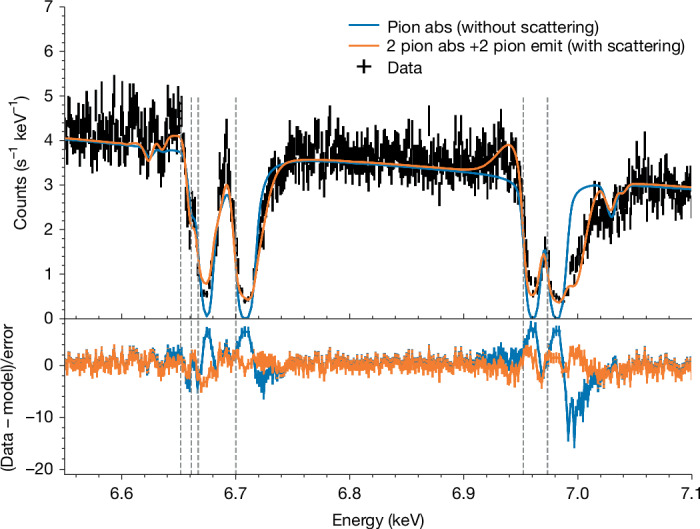
Magnification around the Fe K*α* lines. The dashed vertical lines show the restframe energies of (from left to right) the weak Fe xxiv doublet (6.652 keV and 6.661 keV), Fe xxv (intercombination: 6.667 keV and resonance: 6.700 keV) and the Fe xxvi doublet (6.952 keV and 6.973 keV). The blue line shows a single photoionized absorption model with parameters that fit the multiple narrow lines in the rest of the spectrum. This predicts that the lines are black in their centres, but the data show residual emission due to the presence of diffuse flux (most likely reprocessing and scattering from the wind itself). It also misses the blue wing in the Fe xxvi Lyα_1,2_ absorption line at 7 keV, showing that there is higher velocity material at higher ionization state. The orange line shows our best model, including the additional components in both absorption and emission.

The optically thick column in the slow wind in GX 13+1 is only part of the material obscuring the source. Figure 3 shows a detailed view of the strongest lines, the Fe xxv K*α* intercombination (*x* + *y* = 6.670 keV) and resonance (*w* = 6.700 keV) transitions, and the Fe xxvi Lyα_1,2_ fine structure doublet. The blue line shows the predicted absorption line profiles using the column and velocities derived above for the slow wind, assuming that this covers all of the intrinsic X-ray emission region(s). It is immediately apparent that the model predicts that the narrow cores of these strong lines should be completely black (opaque) at their centres, whereas the data unambiguously show residual flux. This shows there must be an additional source of X-rays that is not absorbed by the wind, most likely the wind itself, which scatters and re-radiates some of the X-rays from the central source, forming a diffuse secondary source of X-rays. The comparison also shows that the single velocity absorption model misses the blue (higher-energy) wing seen especially on the Fe xxvi Lyα_1_ absorption line at around 7 keV, indicating the presence of higher velocity material. This most likely indicates that the wind is stratified, with the most highly ionized material having speeds that are roughly twice that of the slower, less ionized material that forms the narrow line core.

**Fig. 4:**
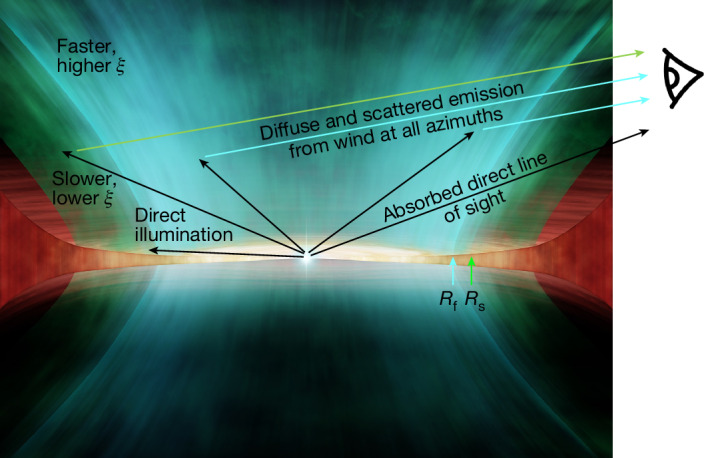
Impression of the wind in GX 13+1 as seen by XRISM. The bulk of the wind (green) is optically thick, highly ionized and slow, but it has a faster, even more highly ionized skin on its inner edge (blue). We see the central source directly through this heavy absorption, but the irradiated wind material forms a secondary source of diffuse X-rays from scattering and re-emission, which can be seen along multiple paths. Illustration by CfA/Melissa Weiss.

Our final model incorporates all these components. We use two absorption zones: a slow absorber to match the bulk of the material and a faster column to match the blue wing on the most highly ionized lines. The model also includes the line and recombination emission calculated by the photoionization code for these two absorption columns to approximate the reprocessed emission from the wind. These emission components are assumed to be at rest but velocity broadened, as expected for emission over all azimuths, and are partially absorbed to approximate the multiple sightlines (Fig. 4). We also include an additional diffuse component from electron scattering of the intrinsic continuum. This is the model (orange) shown with the data in Figs. 1 and 3, with full fit parameters in the Methods (Extended Data Table 2).

The faster wind column density is difficult to robustly constrain. Most of the elements are fully ionized so that there is a large and model-dependent correction between the observed blue wing and the total column. For example, at our best fit (log *ξ* *=* 4.69 ± 0.01), only 10% of Fe is visible as Fe xxvi; all remaining Fe is completely ionized (Methods and Extended Data Fig. 5). The fast wind in our best-fit model has a column density of *N*_H_ = (0.79 ± 0.09) × 10^24^ cm^−2^, increasing the line-of-sight optical depth to *τ*_es_ ≈ 1.8. Correcting for this attenuation results in an even higher estimate for the intrinsic luminosity of *L* ≈ 1.8*L*_Edd_, giving the upper limit of the band of likely intrinsic fluxes shown in blue in Fig. 2.

The two wind components are most likely an approximation of a continuous wind structure because they have similar kinematics. The inner face of the wind (smallest radii) is more highly illuminated and faster, slightly shielding the less ionized, slower material at larger radii. This assumed geometry allows us to estimate the physical parameters of the outflow (Methods). The wind is launched from *R*_f_ ≈ 3 × 10^4^*R*_g_ (6 × 10^9^ cm), with an initial density of about 10^14^ cm^−3^ for a source of intrinsic luminosity 1.8*L*_Edd_. Attenuation by electron scattering reduces the flux to approximately *L*_Edd_ by *R*_s_ ≈ 7 × 10^4^*R*_g_ (1.5 × 10^10^ cm), beyond which the wind is slower. This is shown schematically in Fig. 4.

We can estimate the mass loss rate of the wind if we can independently estimate the solid angle *Ω* subtended by the wind. For optically thin winds, this can be determined from the contribution of scattered emission to the total flux as ${f}_{{\rm{scatt}}}\,\approx (\varOmega /4{\rm{\pi }})(1-{{\rm{e}}}^{-{\tau }_{{\rm{es}}}})$. The observed scattered fraction is difficult to robustly constrain as it depends on the details of how the diffuse flux is modelled. In our fits, the observed scattered fraction ranges from 0.22 to 0.04 (Methods and Extended Data Tables 1 and 2). We assume that these numbers bracket the true scattered flux, so 0.04 < (*Ω*/4π) < 0.22. The assumed mass profile gives a larger uncertainty, and both together give a range in total (fast plus slow) mass loss rate of $1.2 < {\dot{M}}_{18} < 39$, where ${\dot{M}}_{18}$ denotes units of 10^18^ g s^−1^ (Methods). This is approximately 0.3–10 times the inferred mass accretion rate onto the neutron star. This highly non-conservative mass transfer, with as much or more mass being ejected from the system than is accreting, is often seen in galactic binary disk winds^27,28^. Nonetheless, the kinetic power in this wind is very much smaller than the radiative power, as its velocity (even with the faster component) is much less than *c*.

Similarly high-column winds were suggested to explain rare observations of BHB (black hole binary) stars with potentially similar properties (near-Eddington or super-Eddington flux, large disk, high inclination^29–32^). However, without both (1) broadband spectra to show the edge depth as in Fig. 2 and sensitive high-resolution spectra above 7 keV to reveal unsaturated high-order lines (Fig. 1, bottom), it is difficult to distinguish between an intrinsically dim source with an optically thin wind (*N*_H_ ≈ few times 10^23^ cm^−2^) and a source that is much brighter but strongly attenuated by an optically thick wind.

Accretion disk winds in X-ray binaries are often viewed as small-scale versions of the winds from supermassive black holes that drive much of AGN feedback: whether these winds are launched by magnetic fields, radiation pressure or Compton heating remains an open question across the mass scale^1–4^. The XRISM observation of GX 13+1 provides one of the most sensitive probes of the physics of accretion disk winds, to date. We, therefore, consider the origin of this wind by comparing with expectations for winds from all of these driving mechanisms.

The source is at or above the Eddington limit, meaning that radiation from within the disk is strong enough to directly launch its own photosphere as a wind. For *L* ≈ 1 − 2*L*_Edd_, this occurs only in the inner disk, in which the local flux peaks. This Eddington wind should be fast, with mildly relativistic velocities *v*_out_ ≈ 0.2*c* (ref. ^33^), not at all compatible with the observed wind in GX 13+1.

Instead, radiation pressure from the central source could launch a wind by illuminating material at any other radius in the disk, as the effective gravity is proportional to (1 − *L*/*L*_Edd_) (refs. ^34,35^). However, this illumination also heats the disk surface to the radiation temperature. In a sub-Eddington source, this heating alone can be sufficient to drive a wind from radii in which the sound speed exceeds the local escape velocity (called thermal or Compton heated winds)^35–37^. Radiation pressure acts as a boost to the velocity for *L* > 0.2*L*_Edd_ (thermal-radiative winds)^38,39^.

Thermal-radiative driving can give a fairly good match to most previous data on binary winds^2,18,40,41^, but in their simplest form, these models predict narrow lines, as all the material is heated to the same temperature and therefore expands with constant velocity^42^. However, detailed radiation hydrodynamical simulations of thermal-radiative winds from large accretion disks show that these winds start to become stratified at high luminosities (*L* = 0.5–0.7*L*_Edd_) because of optical depth effects. These more realistic simulations of thermal-radiative winds have faster, higher ionization material on the inner, more strongly illuminated face of the wind, with velocities closely matched to those seen in GX 13+1 (refs. ^28,42,43^) (see also refs. ^44,45^). However, the predicted column densities along the line of sight are only a few times 10^23^ cm^−2^, a factor of 10 below those required here. Part of this discrepancy is probably because the simulations do not extend to super-Eddington luminosities, but an additional problem is that current codes do not yet include scattered emission from the wind in calculating the illumination of the disk to launch the wind. This scattered flux can exceed the direct illumination when the wind becomes optically thick^46^.

Alternatively, magnetic winds can also give a stratified velocity and ionization structure. The drawback of this driving mechanism is that the magnetic field configuration (and consequent wind density) cannot now be predicted from first principles, but assuming self-similar, large-scale fields connecting into the disk at all radii^47,48^, gives a wind density structure *n*(*R*) ∝ *R*^−*p*^ with *p* = 1–1.5 (refs. ^49,50^). This predicts that material launched at smaller radii has faster velocity and higher ionization state, generically producing blueward asymmetric line profiles, as observed in the Fe xxvi Lyα_1_ line (Fig. 3). However, this self-similar model is problematic for GX 13+1 as it predicts an additional optical depth of *τ*_es_ ≈ 6 in fully ionized material inwards of the fast component. This would require an uncomfortably large intrinsic source luminosity to produce the observed X-ray flux.

Whatever the physical origin, the observed slow wind can be used to put upper limits on the kinetic power of any fast wind from the inner disk along this line of sight, such as a radiation-pressure-driven wind from the inner, bright *L* ≳ *L*_Edd_ flow. These winds have predicted velocities *v* ~ 0.1–0.2*c* and kinetic power of about 0.05*L*_Edd_ in both analytic and numerical simulations^51–53^, but such high ram pressure material along our sight line would strongly disrupt the slow, quiet kinematics of the observed outer disk wind. Our data require that any fast, wind produced by the *L* ~ *L*_Edd_ inner disk regions must be collimated in the polar direction, potentially by the formation of an inner disk funnel.

This is important as fast, inner disk winds with *v* ≈ 0.2*c* are seen in supermassive black holes, most compellingly from those with *L* ≳ *L*_Edd_ (for example, PDS456; ref. ^5^). This AGN wind was confirmed by recent XRISM data, in which the strong emission as well as absorption signatures require that the wind is quasi-spherical^7^, unlike any inner disk wind in GX 13+1. Understanding this difference in inner disk and wind properties across the mass scale will lead to a deeper understanding of the physics of AGN feedback across cosmic time.

## Methods

### Data extraction

#### XRISM

Data reduction was performed with the software versions of the pre-pipeline version JAXA ‘004_002.15Oct2023_Build7.011’ and the pipeline script ‘03.00.011.008’, and the internal CALDB8, which corresponds to the public XRISM CALDB v.20240815.

The Resolve data were filtered to exclude periods affected by the eclipse of Earth, the sunlit limb of Earth, South Atlantic Anomaly passages, and the initial 4,300 s following the recycling of the 50-mK cooler. Events in the resulting good time intervals were screened using pixel-to-pixel coincidence and an energy-dependent rise time cut^54,55^. Pixels 12 (calibration pixel) and 27 (which shows unexpected gain fluctuations) were excluded. The net exposure time after filtering was 37.8 ks, with a total count rate of 72.1 s^−1^.

A timing coefficient in the CALDB is used to set a flag for any event occurring near-in-time to another event on another pixel. The false coincidence for pixel–pixel coincidence may not be ignored, especially in the bright sources, but in our observations, the loss fraction calculated from the STATUS[4] flag is only about 10%.

Calorimeter events are classified into grades based on the time interval from temporally adjacent events. Here, we use only high-resolution primary (Hp) grade, which provides the highest energy resolution. The Hp count rate was 30.1 s^−1^, representing 42% of the total. A redistribution matrix file was generated using rslmkrmf based on the cleaned event file, with a parameter file of xa_rsl_rmfparam_20190101v006.fits. The line-spread function components considered included the Gaussian core, exponential tail to low energy, escape peaks, silicon fluorescence and electron loss continuum (that is, the ‘X’ option was selected). An auxiliary response file was generated using xaarfgen, assuming a point-like source at the aim point, including the additional opacity of the gate valve closed current configuration of Resolve^56^.

**Extended Data Fig. 1:**
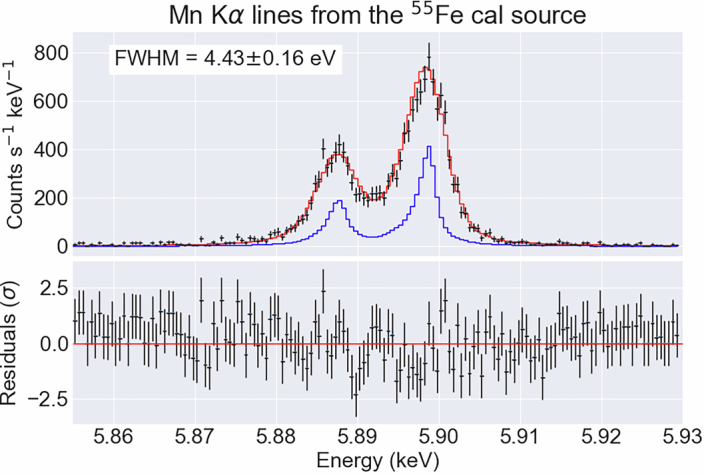
Mn K*α* lines from the ^55^Fe source in the filter wheel. The black bins show the Hp spectrum extracted using two gain fiducial points, summing the 34 pixels. The blue line shows the intrinsic line profile, whereas the red one represents the best fit model, with additional Gaussian broadening of FWHM=4.43 eV. The lower panel shows the residuals between the data and the model, indicating that this is a good description.

**Extended Data Fig. 2:**
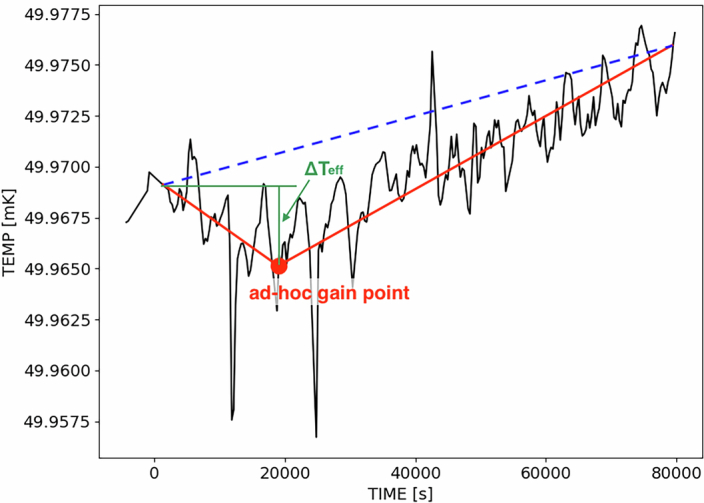
Effective temperature of the calibration pixel versus time. The effective temperature across the observation is shown as a solid black line, compared to a linear interpolation between the measurements at the start and end of the observation (blue dashed line). We introduce an ad-hoc gain point (red filled cicle, with a temperature *ΔT*_eff_ below the first gain point), to give a better match (red solid line).

**Extended Data Fig. 3:**
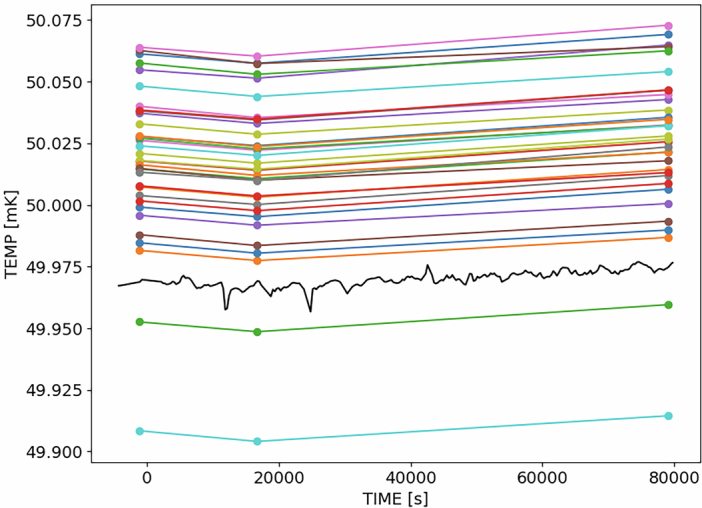
Effective temperature variations in all pixels except 27. Each pixel has an effective temperature estimate corresponding to the gain fiducial measurements at the beginning and end of the observation. We introduced an additional gain point by scaling the ad-hoc gain point from the calibration pixel (see Extended Data Fig. 2) to each individual pixel (see the middle point in each colored line). The black line shows the calibration pixel, which is tracked continuously, for reference.

The temperature sensitivity of the Resolve detector necessitates pixel-by-pixel correction for gain drift to maintain proper energy scale and resolution. The gain scale function is parameterized by an ‘effective temperature’^57^, which was tracked over time for each pixel. The Mn Kα line complex from the ^55^Fe calibration source was used to calculate the effective temperature. Two gain fiducial measurements were performed at the start and end of the observation, in which the entire array was illuminated by the ^55^Fe source in the filter wheel. The Mn Kα spectrum, shown in Extended Data Fig. 1, has an energy resolution of 4.43 ± 0.16 eV (full-width at half maximum) and an energy offset of less than 0.16 eV. A significant temperature shift was identified after the observation, attributed to spacecraft manoeuvres and orientation. In the calibration pixel, continuously illuminated by ^55^Fe, the effective temperature shifted from 49.969 mK to 49.965 mK and then to 49.975 mK (Extended Data Fig. 2). If the gain drift were tracked only at the fiducial points, the maximum effective temperature shift would be 0.005 mK, corresponding to a 1.5-eV energy shift (Extended Data Fig. 2). To reduce this shift, we introduced an ad hoc gain point (Extended Data Fig. 2, red circle) and calculated the effective temperature difference (Δ*T*_eff_) between the initial and ad hoc gain point. Scaling from the gain change on the calibration pixel at this intermediate point, we added a new gain point for each of the other pixels (Extended Data Fig. 3) and corrected the X-ray energies using linear interpolation. After this observation, a gain fiducial 9 h after a manoeuvre was added to standard operations.

At high count rates, energy resolution degradation may occur because of contamination from untriggered electrical cross-talk events^58^. To evaluate this effect, we use spectra of Cr Lyα_1_ and α_2_ lines from the source, which are strong and close in energy to the Mn Kα calibration lines. We compare these before and after cross-talk effect screening, but the line widths do not change significantly. The results indicate that cross-talk has a negligible effect on the energy resolution in this observation. Therefore, we do not apply cross-talk screening to preserve photon statistics.

We also check whether there is contamination of the data from pseudo-Ls (low-resolution secondary) events. However, this is more important for fainter sources and is negligible below 10 keV.

The data are not corrected for any systemic velocity offsets, because these are small, with a blueshift of 40 km s^−1^ for the combined effects of the velocity of the Earth around the Sun and the galactic rotation at GX 13+1 position with respect to the local standard of rest. The peculiar velocity of the binary system is also fairly small, at −70 ± 30 km s^−1^ (ref. ^10^).

#### NuSTAR

We use the nupipeline from HEASOFT v.6.33.2 to reduce NuSTAR observation 30901010002. We use nuproducts to extract NuSTAR source and background spectra and to create response files. The source region is a 1 arcmin circle centred on the source; we used surrounding source-free regions for background.

The NuSTAR observation took place from 25 February 2024 12:56:09 UT to 26 February 2024 10:36:00 UT, and the XRISM observation occurred between 25 February 2024 02:26:51 UT and 26 February 2024 00:06:46. Therefore, to maximize simultaneity and mitigate the effects of any source variability, we define the NuSTAR good time interval as the beginning of the NuSTAR observation to the end of the XRISM observation.

### Ion-by-ion model fitting

Disk-accreting neutron star continuum spectra are generally well modelled by a multicolour disk component, together with higher temperature emission from a boundary layer between the disk and the neutron star surface. The boundary layer illuminates the disk, producing a characteristic reflection spectrum that is broadened by the relativistic velocities and strong gravity of the inner disk. We approximate this component with a broad Gaussian emission line with energy fixed at 6.4 keV. The XRISM spectra are rebinned to require 20 counts per bin and fit between 2.1 keV and 18 keV using the xspec^59^ X-ray spectral fitting package.

Relative to this absorbed disk plus blackbody and Gaussian continuum, the fit residuals show numerous narrow absorption features. We first model these features by considering each ion independently. The kabs model^28^ (a local model for use in xspec software) calculates the full Voigt absorption line profile for a single transition in a given ion. Modelling the full series of transitions from *n* = 1 for a given ion then involves multiple KABS components, with the ion column and velocity outflow and width tied across all the components. We develop a more convenient xspec local model, Ionabs, which packages all these together, calculating the full line series from a given ion column with given kinematics in a single model component. This includes all fine structure lines, as well as the self-consistent edge structure(s) (including the L-shell edge for ions with three or more electrons, such as Fe xxiv). The Voigt profile velocity width in KABS is defined as *v*_turb_/*c* = (*E* − *E*_0_)/Δ*E*, but the photoionization code pion (see below) uses the Gaussian width ${v}_{{\rm{rms}}}/c=(E-{E}_{0})/(\,\sqrt{2}\Delta E)$, so here we report *v*_rms_ so that these can be directly compared. Transition energies, oscillator strengths, Einstein A values and the energy dependence of the cross-sections are taken from Flexible Atomic Code^60^. These match very well with the NIST database for H- and He-like ions.

Most of the lines have a similar kinematic structure with slow outflow velocity and a very narrow velocity width. This ‘slow’ wind component must have a very high column density to produce the multiple higher-order lines (transitions beyond *n* = 8) of Fe xxv and Fe xxvi. This column should have corresponding *n* = 2–1 Kα transitions, which are completely saturated, so that the line cores are completely black. The fact that the data never go to zero shows that there is an additional diffuse source of X-rays, most likely from the wind itself (Fig. 3). Moreover, the detailed Fe xxvi Kα_1_,_2_ line profile shows a strong blue wing, requiring that there is an additional, faster wind component present (Fig. 3).

Thus, we model the intrinsic spectrum absorbed by two wind components: slow (16 ions) and fast (only Fe xxv, Fe xxvi, Ni xxvii and Ni xxviii). Modelling the diffuse emission is more challenging, as it should be extremely complex, with recombination radiation from the X-ray heated material and scattered incident continuum forming a spatially extended source that is absorbed along multiple different lines of sight through the wind. We first approximate this as electron scattering alone, so a fraction *f*_scatt_ of the incident continuum, but a better fit to the remaining residuals around the absorption lines is if the scattered continuum is also absorbed by the fast wind component.

The model then consists of the intrinsic continuum Int=(diskbb+gauss+bbody), absorbed by multiple ions grouped into two kinematic components (slow: Ionabs_s_ and fast: Ionabs_f_), together with photoelectric absorption from neutral material (TBabs) fixed at the interstellar column density of *N*_H_ = 3.2 × 10^22^ cm^−2^. The model is ${\rm{TBabs}}\times ({{\rm{Ionabs}}}_{{\rm{s}}}^{{\rm{16}}}\,{{\rm{Ionabs}}}_{{\rm{f}}}^{{\rm{4}}}+{{\rm{f}}}_{{\rm{scatt}}}\,{{\rm{Ionabs}}}_{{\rm{f}}}^{{\rm{4}}})\times {\rm{Int}}$ in XSPEC notation, in which the superscripts on Ionabs show the number of ions included.

The resulting fit parameters are given in Extended Data Table 1. The ions in the slow component all have similar outflow velocities of *v*_out_ ~ 330 km s^−1^. The fast component seems to have a wider range of kinematics, with outflow velocities ranging from about 500–1,000 km s^−1^ depending on the ion, but both fast and slow components have line widths of around *v*_out_/2.

The column densities derived for the ions in the slow component (Extended Data Table 1) are almost an order of magnitude larger than found in previous Chandra or HETGS data (ref. ^15^). We estimate a lower limit to the equivalent hydrogen column density from adding the slow component Fe xxv and Fe xxvi ion columns together, to get ${N}_{{\rm{Fe}}}=4{6}_{-5}^{+6}\times 1{0}^{18}\,{{\rm{cm}}}^{-2}$, giving *N*_H_ > *N*_Fe_/*A*_Fe_ = 1.4 ± 0.2 × 10^24^ cm^−2^ for *A*_Fe_ = 3.3 × 10^−5^.

This column density is large enough that electron scattering optical depth is significant: *τ*_es_ = 1.21*N*_H_*σ*_T_ ≳ 1.1, where the factor 1.21 comes from the number of electrons per hydrogen atom in a fully ionized plasma of solar abundance and *σ*_T_ is the Thomson cross-section. The observed Fe ion columns in the slow wind component already imply that the wind is optically thick to electron scattering, and yet there should be even more material, first because of the fast wind and second because some fraction of Fe is completely stripped to Fe xxvii, and hence produces no line signatures. This correction need not be very large for the slow wind, for which the ratio of columns Fe xxvi/Fe xxv is close to unity. However, this is not true for the fast wind, for which the column in Fe xxvi is 2.5 times larger than that of Fe xxv. Thus, the observed ion columns in the fast wind are probably only a small tracer of the likely column present. To correct for this, an ionization balance calculation needs to be done.

### Photoionization modelling for ion fractions

**Extended Data Fig. 4:**
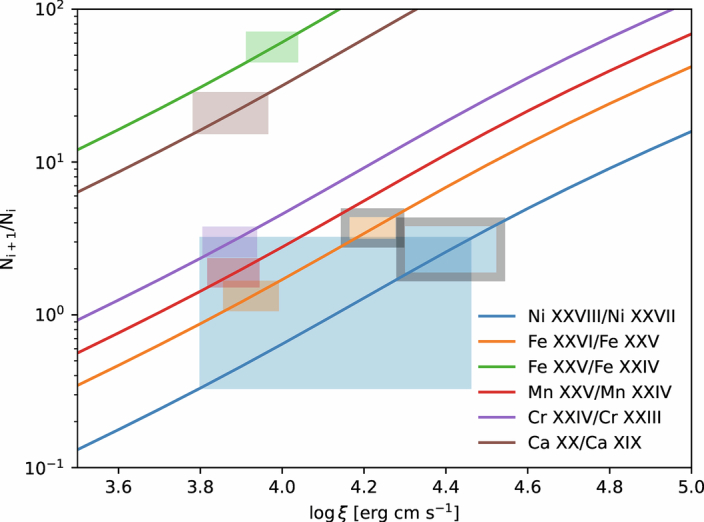
Ion ratio as a function of ionisation parameter. We computed the ground state populations for each ion using the pion code as in Methods. The ratio of these populations (equivalently, the ratio of the column densities in different ions) is sensitive to the ionisation parameter, as shown. Using the ratio of column densities taken from Extended Data Table 1, we estimate the ionisation parameter of the slow component in our ion-by-ion fits as $\log \,\xi  \sim 3.9$ (shaded regions), and the fast component of Fe and Ni as $\log \,\xi =4.15-4.53$ (shaded regions with black frames).

**Extended Data Fig. 5:**
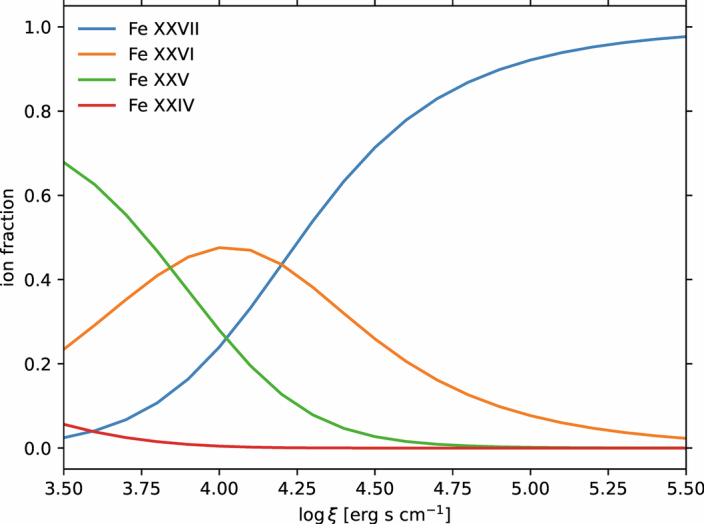
Ion fractions of Fe versus the ionisation parameter. This is computed using pion as described in Methods, assuming that the gas is photoionised by the continuum shape observed. We estimate the ionisation parameter from our ion-by-ion fits using Extended Data Fig. 4, then used the curves above to determine the column density of completely-ionised Iron (Fe xxvii).

We use the photoionized plasma model pion v.3.08.00, available in SPEX (refs. ^23,61^). We compute the ratios of different ionization stages *N*^*i*+1^/*N*^*i*^ (Extended Data Fig. 4) and the ion fractions (Extended Data Fig. 5) as functions of the ionization parameter log *ξ* *≡* *L*/(*nR*^2^). We assume an intrinsic illuminating continuum that matches the best-fit incident continuum (disk blackbody plus blackbody).

All the ion ratios in the slow component are consistent with those from Fe xxvi/Fe xxv alone, giving an ionization parameter of log *ξ* = 3.85–3.98. This gives an ion fraction for completely ionized iron (Fe xxvii) of *f*_xxvii_ = 0.14–0.23 (Extended Data Fig. 5). This increases the total iron column density in the slow wind to *N*_Fe_ = 51–60 × 10^18^ cm^−2^, leading to an equivalent hydrogen column density *N*_H_ = *N*_Fe_/*A*_Fe_ = 1.5–1.8 × 10^24^ cm^−2^ and *τ*_es_ = 1.2–1.4.

A similar analysis for the fast wind gives a more complex picture. The Fe ratio suggests log *ξ * = 4.15–4.29 (corresponding to ion fraction of Fe xxvii 0.38–0.53), giving *N*_H_ ~ 4.3–5.6 × 10^22^ cm^−2^. However, these broader lines are less well-defined in the data, and therefore more sensitive to the model assumed to approximate the complex diffuse emission from the wind. Thus, the column density of the faster component is much more uncertain (see the full photoionization fits below).

The unabsorbed continuum model (without scattered flux) gives a bolometric flux of *F* = 9.1 × 10^−9^ erg cm^−2^ s^−1^ (13.6–100 keV). Correcting this for attenuation by electron scattering with *τ*_es_ = 1.2–1.4 gives an intrinsic flux of *F*_0_ = 3.0–3.8 × 10^−8^ erg cm^−2^ s^−1^. The luminosity of this source *L* = 4π*d*^2^*F*_0_ = 0.8 − 1*L*_Edd_, where *d* = 7 kpc and *L*_Edd_ = 2.1 × 10^38^ erg s^−1^.

The scattered fraction parameter in these fits *f*_scatt_ is the ratio of scattered to observed direct flux. We use this to calculate the ratio of scattered to intrinsic flux *F*_scatt_/*F*_0_ = 0.052–0.066 and use this to estimate the solid angle of the wind, as *F*_scatt_/*F*_0_ ≈ *Ω*/4π(1 − exp(−*τ*_es_)). This gives *Ω*/4π = 0.08 ± 0.01, although again this is quite uncertain as it depends on the detailed wind geometry and emission or absorption.

### Fitting with photoionization models

We now use the same photoionized code, PION, to directly fit to the data. We calculate a grid of models for solar abundances, simulating absorbers with different values of column density *N*_H_, ionization parameter log *ξ* and turbulent velocity *v*_rms_, fixing the illuminating SED shape to that derived from spectral fitting. Each of the simulations has 65,536 logarithmically spaced bins to cover the energy range from 10^−4^ keV to 10^3^ keV with a resolution of 1.5 eV around 6 keV, enough to fit Resolve data. In total, we perform 8,736 simulations with values 21 ≤ log *N*_H_ ≤ 25 spaced by 0.2 (21 points), 2 ≤ log *ξ* ≤ 7 spaced by 0.2 (26 points), and −5 ≤ log(*v*_rms_/*c*) ≤ −2 spaced by 0.2 (16 grid points). We fit with a single number density *n*_*p*_ = 10^14^ cm^−3^ to reduce the size of the tables (see the main text and below). We calculate the population levels from radiative recombination, cascade, radiative and collisional excitation correctly for meta-stable levels^42,62^. We build these results into a multiplicative absorption table model^46^ for use in XSPEC.

#### Diffuse continuum approximated by absorbed scattered flux

We replace the multiple ion-by-ion absorption components with two pion absorption components (one fast: abs_f_ and the other slow: abs_s_). As above, we assume that the diffuse emission has the same shape as the incident continuum and that this scattered component is absorbed by the fast wind. We represent this model in XSPEC form as TBabs × (abs_s_abs_f_Int + *f*_scatt_abs_f_Int).

The goodness of the fit is not far from that of the ion-by-ion fit, which allowed free element abundances and allowed every ion to have different kinematics and ion ratios (*χ*^2^ = 14,726/13,555, that is, 52 fewer free parameters, for Δ*χ*^2^ ~ 550). This gives very similar plasma parameters for the slow component as derived from the ion-by-ion fitting, namely, $\log \,\xi =3.9{3}_{-0.02}^{+0.01}$ and ${N}_{{\rm{H}}}=15{4}_{-6}^{+8}\times 1{0}^{22}\,{{\rm{cm}}}^{-2}$. However, the fast component now has a higher ionization parameter (log *ξ* = 4.49) and hence a higher equivalent column density of ${N}_{{\rm{H}}}={9}_{-1}^{+2}\times 1{0}^{22}\,{{\rm{cm}}}^{-2}$. This shows that the fast component parameters are more sensitive to the details of the model, but that the slow component is very robust, and robustly gives an optical depth *τ*_es_ > 1.

#### Diffuse component, including line and recombination emission

A better approximation to the diffuse and scattered continuum requires using pion to calculate the emitted line and recombination continua from the photoionized material, apart from the absorption lines. We take the same incident spectrum and density as for the absorption table model to generate an additive table model for XSPEC (hereafter emm), but this time we set the solid angle fraction *Ω*/4π = 1.0. In principle, the resulting emission normalizations allow the solid angle to be independently estimated, but these are dependent on the details of the radiation transfer through the optically thick wind, so we do not use them here.

We tie the ionization parameter and column density to be the same for the absorbing and emitting plasma. We expect that the emission should arise from all azimuths, so we fix the outflow velocity to zero and allow the broadening to be free. We allow this to be self-absorbed by the wind in our line of sight, but caution that this is just an approximation to a more complex geometry that requires a full radiation transfer calculation.

The final model is TBabs × (abs_s_abs_f_Int + f_scatt_abs_f_Int + abs_s_emm_f_ + f_scatt_emm_f_ + emm_s_), where again we fix TBabs to the interstellar column density of *N*_H_ = 3.2 × 10^22^ cm^−2^. This gives our best description of the spectrum (Extended Data Table 2), and is the model shown in Fig. 1. This gives a goodness of fit of 14,339/13,551. This has four more free parameters than the previous description of the diffuse flux, but gives Δ*χ*^2^ = −386. It is now statistically equivalent to the ion-by-ion fits in Extended Data Table 1, because it has 48 fewer free parameters for an increase of Δ*χ*^2^ = +151.

It is difficult to see the emission lines in this model because they are dominated by the fast component and are therefore moderately broadened. Nonetheless, the resultant P Cygni profile can be seen in Fe xxvi Lyα_1,2_ at around 6.94 keV as shown in Fig. 3. The emission lines also contribute to the shape of the saturated line cores, and this more complex model shifts the ionization parameter of the fast wind to even higher values, $\log \,\xi =4.6{9}_{-0.04}^{+0.03}$ requiring even higher column densities: *N*_H_ = 80 × 10^22^ cm^−2^. Again, the parameters of the slow wind are mostly consistent with previous models, with just slightly lower column (${N}_{{\rm{H}}}=13{2}_{-8}^{+7}\times 1{0}^{22}$ cm^−2^) and ionization state (log* ξ* = 3.88 ± 0.01), but similar kinematics and scattered fraction.

We also perform fits with the XSTAR model warmabs^63^ to explore the overall robustness of our photoionization analysis. For warmabs, we calculate electron-level populations using the best-fit continuum from fits to a model consisting of an absorbed disk plus nthcomp^64,65^ with Gaussian lines and a smeared edge near 8 keV. Fits to the Resolve spectrum with warmabs were qualitatively and quantitatively very similar to the pion fits, requiring a high-column component with a smaller turbulent line width and blueshift and a more highly ionized, broader and faster component. These fits will be presented in detail elsewhere, but we note that despite different assumptions about the ionizing continuum, radiative transfer, absorption and emission geometry, and the different codes, we still find a total equivalent of the slow wind column density in excess of 1.4 × 10^24^ cm^−2^.

### Wind geometry

We assume that the wind is a continuous structure so the outer edge of the fast wind must coincide with the inner edge of the slow wind^32^. In other words, the column density of the fast and slow winds must be given by

$${N}_{{\rm{H}},{\rm{f}}}={\int }_{{R}_{{\rm{f}}}}^{{R}_{{\rm{s}}}}n(R){\rm{d}}R,\quad {N}_{{\rm{H}},{\rm{s}}}={\int }_{{R}_{{\rm{s}}}}^{{R}_{{\rm{out}}}}n(R){\rm{d}}R,$$

where *R*_s_ = *R*_f_ + Δ*R*_f_, Δ*R*_f_ is the width of the fast wind and *n*(*R*) is the density profile of the wind. But the relative locations of the fast and slow winds are also set by their relative ionization parameters. If the ionization parameter at the inner edge of the fast wind is ${\xi }_{{\rm{f}}}={L}_{0}/{n}_{{\rm{f}}}{R}_{{\rm{f}}}^{2}$, then the ionization parameter at the inner edge of the slow wind must be ${\xi }_{{\rm{s}}}={L}_{0}\exp (-{\tau }_{{\rm{f}}})/{n}_{{\rm{s}}}{R}_{{\rm{s}}}^{2}$. Here *n*_f_ and *n*_s_ are the densities of the wind at *R*_f_ and *R*_s_, respectively, whereas *R*_out_ is the outermost radius at which the wind is produced (which need not be the same as the disk outer radius). The factor exp(−*τ*_f_) is an approximation of the attenuation of the radiation field by the fast wind. This is appropriate for a relatively small solid-angle wind, as inferred here.

For a self-consistent solution, the radius of the slow wind as inferred from the column density of the fast wind must match the radius implied by the relative ionization of the two zones. This gives four independent constraints, so we can solve for (at most) four independent parameters. We first assume a constant density wind, which gives *n*_f_ = *n*_s_ = 1.6 × 10^14^ cm^−3^, for *R*_f_ = 4.7 × 10^9^ cm, *R*_s_ = 1.0 × 10^10^ cm and *R*_out_ = 1.8 × 10^10^ cm. Alternatively, we assume a power law density distribution $n={n}_{{\rm{f}}}{(R/{R}_{{\rm{f}}})}^{-x}$ for *R*_f_ < *R* < *R*_out_ = 10^12^ cm, which is the outer disk radius. This has *n*_f_ = 0.9 × 10^14^ cm^−3^ with *x* = 1.1 for *R*_f_ = 6.3 × 10^9^ cm, *R*_s_ = 3.7 × 10^10^ cm (giving *n*_s_ = *n*(*R*_s_) = 1.3 × 10^13^ cm^−3^).

These densities are very high, but the predominantly H- and He-like ions seen have no density diagnostic potential. Instead, previous work on the black hole binary GRO J1655-40, which also showed evidence for a Compton-thick wind from a likely super-Eddington state^30,42,66,67^, measured density directly from a meta-stable L-shell absorption line of B-like Fe xxii (refs. ^42,68,69^). This line transition at about 1 keV is outside of the current Resolve bandpass, and would probably not be present in the higher ionization state seen in the GX 13+1 outflow. However, weak meta-stable lines from K-shell Be-like Fe xxiii around 6.6 keV may be used to probe the density^62^ in future modelling.

In principle, a thermal wind may be launched from all radii *R* ≳ 0.2*R*_IC_, where *R*_IC_ is the Compton radius^37^. For GX 13+1, this nominal limit is approximately 3.3 × 10^10^ cm, a factor of a few larger than the inferred launch radius of the fast wind. However, thermal-radiative winds can be expected from much smaller radii when the luminosity approaches the Eddington limit^35^, so our radii are probably consistent with a thermal-radiative wind.

Finally, we calculate the mass loss rate in the wind. Here the wind is being launched from all radii on the disk from *R*_f_ − *R*_out_, so we cannot use the standard mass continuity expressions as the wind mass is increasing over this range. Instead, we calculate the total wind mass in this region, *M*, and the time, *t*, it takes to expand out of this region as

$$\begin{array}{c}M={\int }_{{R}_{{\rm{f}}}}^{{R}_{{\rm{out}}}}4{\rm{\pi }}{R}^{2}(\varOmega /4{\rm{\pi }})n(R)m\,{\rm{d}}R,\\ t={\int }_{{R}_{{\rm{f}}}}^{{R}_{{\rm{out}}}}\frac{{\rm{d}}R}{v(R)}=\frac{{R}_{{\rm{s}}}-{R}_{{\rm{f}}}}{{v}_{{\rm{f}}}}+\frac{{R}_{{\rm{out}}}-{R}_{{\rm{s}}}}{{v}_{{\rm{s}}}}\end{array}$$

where *m* = 2.4 × 10^−24^ g is the average atomic mass per hydrogen atom in a cosmic gas, *Ω* is the solid angle of the wind and *v*(*R*) is the radial velocity profile.

For the constant density wind, these give $\dot{M}=M/t=2.4-6.6\,\times$ $1{0}^{18}\,{\rm{g}}\,{{\rm{s}}}^{-1}$ for the solid angles discussed in the main text (0.08 ≤ *Ω*/4π ≤ 0.22). This is very similar to the estimates given by mass continuity $\dot{M}=4{\rm{\pi }}{R}^{2}n(R)v(R)m(\varOmega /4{\rm{\pi }})$, which can be rewritten for the fast wind as ${\dot{M}}_{{\rm{f}}}=4{\rm{\pi }}m(\varOmega /4{\rm{\pi }})(L/{\xi }_{{\rm{f}}})=0.5-1.5\times 1{0}^{18}\,{\rm{g}}\,{{\rm{s}}}^{-1}$, whereas the slow wind gives ${\dot{M}}_{{\rm{f}}}=4{\rm{\pi }}m(\varOmega /4{\rm{\pi }})L\exp (-{\tau }_{{\rm{f}}})/{\xi }_{{\rm{s}}}=1.3-3.7\times 1{0}^{18}\,{\rm{g}}\,{{\rm{s}}}^{-1}$. However, the power law density profile with *x* = 1.1 has much more mass at larger radii, so it gives much larger $\dot{M}=M/t=14-39\times 1{0}^{18}\,{\rm{g}}\,{{\rm{s}}}^{-1}$.

Even the lowest estimates for the mass loss rate from constant density assumptions are comparable with the central mass accretion rate of 3.9 × 10^18^ g s^−1^ required to power the inferred X-ray luminosity, whereas the largest estimates have up to 10 times more matter ejected than is accreted.

## Online content

Any methods, additional references, Nature Portfolio reporting summaries, source data, extended data, supplementary information, acknowledgements, peer review information; details of author contributions and competing interests; and statements of data and code availability are available at 10.1038/s41586-025-09495-w.

## Figures and Tables

**Extended Data Table 1:**
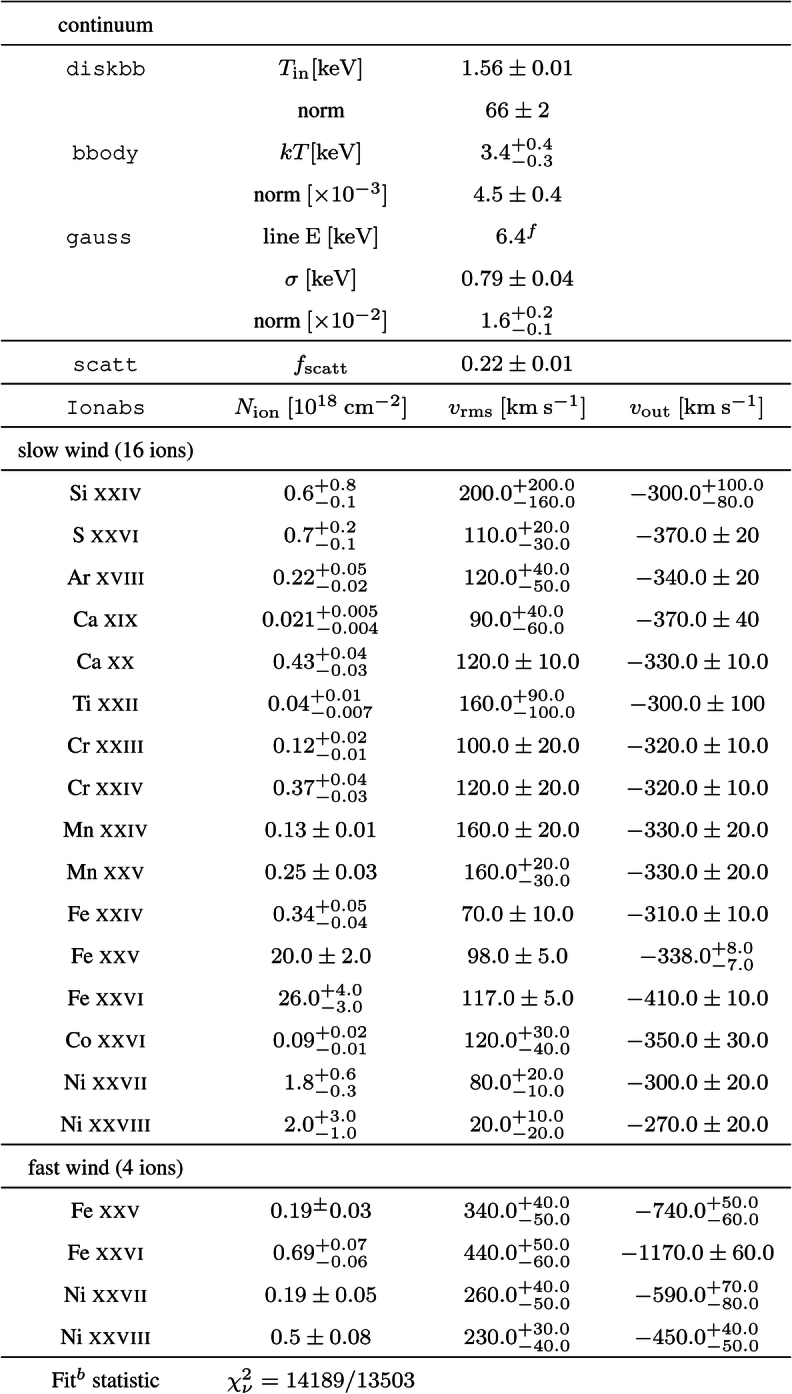
Fit with ion-by-ion absorption plus scattered flux

^*a*^*N*_ion,18_ is the ion column density in units of 10^18^ cm^−2^. ^*b*^Model is ${\mathtt{TBabs}}\times ({{\mathtt{Ionabs}}}_{{\rm{s}}}^{{\mathtt{16}}}\,{{\mathtt{Ionabs}}}_{{\rm{f}}}^{{\mathtt{4}}}+{{\mathtt{f}}}_{{\rm{scatt}}}\,{{\mathtt{Ionabs}}}_{{\rm{f}}}^{4})\times {\mathtt{Int}}$. ^*f*^indicates fixed parameters. All other parameter errors are calculated at 90% confidence levels.

**Extended Data Table 2:**
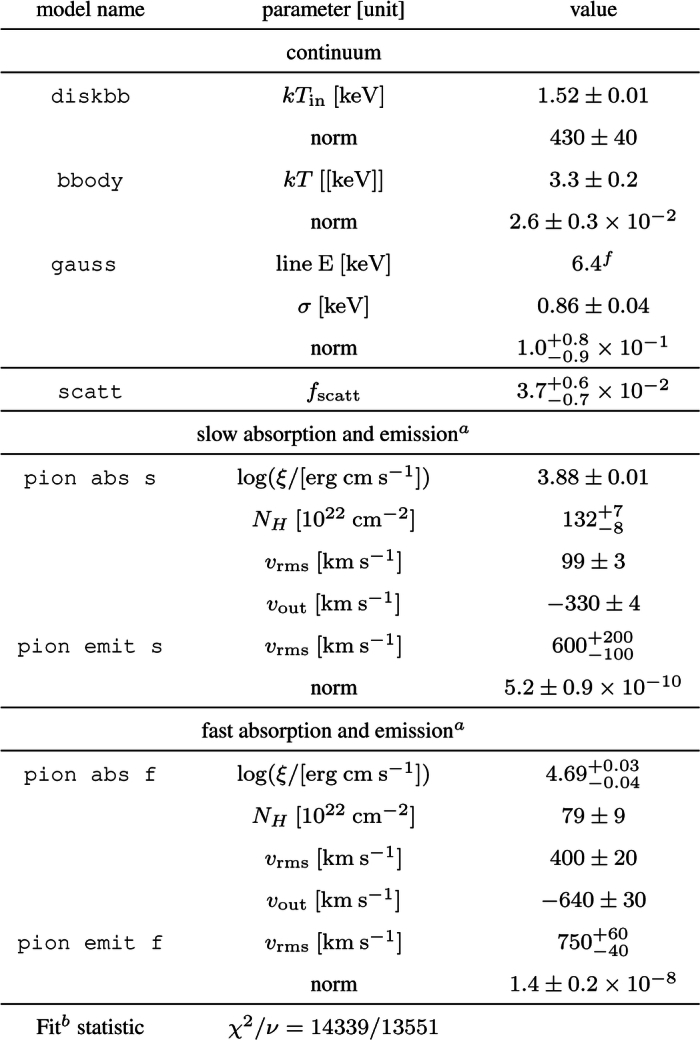
Fit with two pion absorbers plus their emission and scattered flux

^*a*^The column density and ionisation state are tied between the absorption and emission. ^*b*^model is TBabs × (abs_s_abs_f_ Int + f_scatt_ abs_f_ Int + abs_s_ emm_f_ + f_scatt_ emm_f_ + emm_s_). ^*f*^indicates fixed parameters. All other parameter errors are calculated at 90% confidence levels.

## Data Availability

The XRISM Resolve data will be publicly available in the archives after the proprietary period ends. The NuSTAR dataset (ObsID 30901010002) is already publicly available.

## Code availability

The pion photoionization code is publicly available as part of the spex package. The warmabs photoionization code is publicly available as part of the xstar package. The ionabs code is publicly available for download at GitHub (https://github.com/ryotatomaru/Ionabs) as a local model for installation and use in the xspec package. The XSPEC model files used to make Extended Data Table 2, including the PION tables, are publicly available for download at Zenodo^70^ (10.5281/zenodo.15628497).
